# Universal Testing and Treatment as an HIV Prevention Strategy: Research Questions and Methods

**DOI:** 10.2174/157016211798038515

**Published:** 2011-09

**Authors:** Richard Hayes, Kalpana Sabapathy, Sarah Fidler

**Affiliations:** 1 MRC Tropical Epidemiology Group, London School of Hygiene & Tropical Medicine, London, UK; 2 Faculty of Medicine, Imperial College London, UK

**Keywords:** HAART, highly active antiretroviral therapy, HIV prevention, randomized controlled trials.

## Abstract

Achieving high coverage of antiretroviral treatment (ART) in resource-poor settings will become increasingly difficult unless HIV incidence can be reduced substantially. Universal voluntary counselling and testing followed by immediate initiation of ART for all those diagnosed HIV-positive (universal testing and treatment, UTT) has the potential to reduce HIV incidence dramatically but would be very challenging and costly to deliver in the short term. Early modelling work in this field has been criticised for making unduly optimistic assumptions about the uptake and coverage of interventions. In future work, it is important that model parameters are realistic and based where possible on empirical data. Rigorous research evidence is needed before the UTT approach could be considered for wide-scale implementation. This paper reviews the main areas that need to be explored. We consider in turn research questions related to the provision of services for universal testing, services for immediate treatment of HIV-positives and the population-level impact of UTT, and the research methods that could be used to address these questions. Ideally, initial feasibility studies should be carried out to investigate the acceptability, feasibility and uptake of UTT services. If these studies produce promising results, there would be a strong case for a cluster-randomised trial to measure the impact of a UTT intervention on HIV incidence, and we consider the main design features of such a trial.

## INTRODUCTION

Despite impressive advances in the provision of antiretroviral therapy (ART) for HIV-infected individuals in resource-poor settings based on current treatment guidelines, only around one-third of the 15 million individuals who are eligible for treatment are currently receiving it [1]. Furthermore, for each patient started on ART, an additional two individuals become newly infected with HIV [1]. Unless the incidence of new HIV infections can be reduced, there will be a continuous increase in the number of HIV-positive individuals who will require ART in future. Achieving high coverage of ART will therefore become increasingly challenging in the absence of substantial reductions in HIV incidence.

Unfortunately, there are very few proven tools for HIV prevention. A review of randomised controlled trials (RCTs) of HIV prevention methods found that of 39 interventions tested in rigorously conducted RCTs, only 5 showed significant evidence of protection [2]. These included the three trials of male circumcision [3-5], the Mwanza trial of sexually transmitted infection (STI) treatment for HIV prevention [6] and the *RV144* trial of a prime-boost vaccine regimen in Thailand [7]. Male circumcision reduces female-to-male HIV transmission by 50-60%, but wide-scale implementation of safe services for male circumcision has been slow. The vaccine trial was of borderline significance and showed a modest effect, while STI treatment as an HIV prevention measure seems to be relatively less effective in the mature epidemics which now predominate in sub-Saharan Africa [8].

The results of the *Centre for the AIDS Programme of Research in South Africa (CAPRISA) 004* trial of tenofovir gel, showing a 39% impact on HIV incidence, have given renewed hope to the microbicide field [9]. The *Preexposure Prophylaxis Initiative (iPrEx)* trial of pre-exposure prophylaxis (PrEP) for men having sex with men (MSM) gave further hope for using antiretrovirals for prevention, with a 41% reduction in HIV incidence in those receiving an oral combination of daily tenofovir/emtricitabine [10]. Unfortunately a similar placebo controlled trial of PrEP in African women (*FEM-PrEP*) was discontinued early when it was deemed unlikely that the intervention could show a protective effect in the study population [11]. Further trials of ART-based microbicides as well as oral regimens for use as PrEP are in progress and will report in the next five years^*^.

Given the limited array of proven prevention tools, universal testing and treatment (UTT) has been proposed as a potential new HIV prevention strategy that may be highly effective in high prevalence settings [12-17]. UTT would involve offering HIV voluntary counselling and testing (VCT) to the entire population, and offering immediate ART to all those testing HIV-positive irrespective of clinical stage or CD4 count. Mathematical modelling has shown that UTT could lead to steep reductions in HIV incidence and might potentially eliminate HIV as a public health problem over a period of 15-20 years, as well as reducing HIV-related morbidity and mortality [12-16,18-20]. These projections are based on assumptions that have been questioned, however, and some have argued that the likely effects of a UTT intervention would be much more limited [21,22]. Furthermore, implementation of UTT on a wide scale would be logistically and financially challenging, and might overwhelm existing health services.

A trial that was recently halted early, due to unequivocal evidence of a protective effect of early ART, currently provides the most persuasive data to support universal treatment to prevent transmission. The *HIV Prevention Trials Network (HPTN) 052* study included 1700 discordant couples (97% heterosexual) in which one partner was infected with HIV and had a CD4 count between 350 and 550 cells/mm^3^, from 13 sites in Africa, Asia, and the Americas [23]. Only one case of HIV transmission occurred among the couples assigned to receive immediate treatment (median CD4 count at entry was 436 cells/mm^3^) compared to 27 cases among those in the delayed treatment arm (treatment onset at CD4 count <250 cells/mm^3^), representing a 96% reduction in HIV transmission to the uninfected partner.

Before UTT is considered for implementation on a wide scale, it is important that rigorous evidence is collected on the feasibility and effectiveness of the UTT strategy, and on any adverse effects of the intervention including toxicity or the development of ART resistance. In this paper, we discuss the main research questions that need to be addressed in collecting this evidence and the research methods that could be used to answer them. We begin by discussing research issues related to the provision of universal VCT before going on to explore issues in the provision of immediate treatment and care for all those diagnosed HIV-positive. We then consider research to determine the effects on HIV transmission at individual and population levels. We conclude that a large-scale cluster-randomised trial would be needed to measure the impact of UTT on HIV incidence at population level, and briefly discuss some of the design issues in implementing such a trial. We focus here on research on UTT in sub-Saharan Africa, where there is the most urgent need for effective HIV control.

## UNIVERSAL HIV COUNSELLING AND TESTING

### Introduction

Awareness of HIV status is the first step in accessing care, and has been strongly advocated not only as a potential prevention tool but also as a way to normalise and destigmatise HIV [24]. However, it is estimated that less than a quarter of 15-49 year olds in sub-Saharan Africa know their HIV status [1]. In order to make UTT achievable, a better understanding of the barriers to testing, knowledge of test result and subsequent linkage with care must be gained to make important operational changes to service provision.

In recent years, progress in extending coverage and uptake has been made through provision of vertical and integrated services, client- and provider-initiated HIV counselling and testing, opt-in and opt-out approaches and other innovative methods such as home-based and work-place testing [25-30]. These achievements have to be built upon for UTT, but operational research is required to examine the acceptability, effectiveness, feasibility and sustainability of these approaches. Mixed methods research, combining qualitative and quantitative approaches, will be needed to provide insights to patient behaviour [31]. Findings from studies done in the current context of relatively low access to testing and treatment, and with ART reserved for those with advanced immune suppression, may not be generalisable to a future UTT model where individuals without significant HIV-related illness are invited to initiate ART. Therefore, questions already examined in existing contexts may need to be re-examined in the context of UTT.

In this section, we discuss research questions relating to models of service provision, promotion of universal testing, methods and frequency of testing, counselling strategies, uptake, community-level response and costs. Key research questions and potential research methods are summarised in Table **1**.

### Models of Service Provision

The most suitable model of service provision may vary depending on the context and setting where UTT is to be implemented. Nonetheless, when developing a model of care, there are key questions that would be relevant for any setting. Various models of VCT provision have been used to date, and it appears that ease of access to testing and receiving a result is the most critical determinant of uptake. Work-place and home-based testing have been shown to be advantageous [25,30,32], as has community-based VCT provision when compared to standard clinic-based provision [33]. However, questions remain concerning how well these modes of testing will lead to linkage with treatment and care; what are the implications with respect to confidentiality, stigma or pressure to accept a test; and the sustainability of such work-place and home-based services in the long term given numerous competing priorities and pressures on resources.

A variety of models will need to be examined, including door-to-door campaigns and home-based testing; work-place VCT services; mobile test units that visit communities periodically; stand-alone VCT centres in conveniently placed communal locations (e.g. market-places, bus-stands, religious venues); or on-site VCT within health units, provided either as a stand-alone service or integrated within healthcare provision in an “opt-out” model. It is important to examine multiple options as there will be population sub-groups to whom some models will not be available or acceptable, and alternatives should exist if high uptake is to be achieved. Additionally, VCT provision models should consider extending services provided through UTT to ensure access to proven prevention tools such as male circumcision, condoms or microbicides (if licensed in the future). Standardised client identification and record keeping will be necessary to co-ordinate testing efforts and ensure more accurate analysis of attendance across testing and care sites, as duplication may be a problem. Qualitative research with users and non-users of different types of services, as well as service providers, will provide useful insights.

### Staff Requirements for HIV VCT

Given the severe human resource shortages in many sub-Saharan African countries, task-shifting has become the mainstay of HIV care and perhaps most widely in provision of VCT. Yet the evidence regarding task-shifting in VCT is relatively limited [34]. The minimal cadre of health care worker required to deliver an adequate level of service and the number of trained staff and other resources required to carry out universal testing need further exploration. Community health workers, lay counsellors and even “expert patients” may provide the most feasible option for roll-out of UTT. When assessing these questions, the quantity and quality of service provided by a given model of testing needs to be examined. Operational research is needed to examine the volume of clients that a given cadre of staff is able to see, the accuracy of results given (by comparing VCT results against gold standard laboratory testing) and client satisfaction with the quality of service provided.

Self-testing using oral swabs [35,36] and oral fluid test kits has been explored as a model to increase knowledge of HIV status. Encouraging results were found in a preliminary study in Malawi, where 95% of adults selected for study were seen, and 92% of these opted for self-testing [37]. The validity of their test results was shown to be close to 100%. While this approach may be controversial, it may provide an additional method which is discreet, easy and non-invasive to perform and appealing to those who are reluctant to use other services and may encourage repeat testing. Care will be needed to ensure that patients who test HIV-positive are linked to services for provision of counselling and treatment.

Integrating HIV testing into the care of patients presenting with conditions such as tuberculosis (TB) and in antenatal care facilities has been successful [38,39], but the offer of an HIV test to *any* individual engaging with a health care facility in an expanded provider-initiated testing model (opt-out testing for all clinic and hospital patients) has yet to be assessed [40,41]. This could be an important opportunity to capture missed diagnoses especially as HIV may be the underlying cause of a given presentation for medical care.

### Promotion of Universal Testing

Before embarking on a UTT programme, understanding should be gained of likely community perceptions of UTT and how best to promote UTT in a wide-reaching, simple and clear way [42].

Imperative in exploring community perceptions are questions relating to what concerns exist over UTT. Would there be fear of forced testing or abuse of test results? Would there be mistrust of UTT and its purpose as a public health prevention tool, given that treatment has previously been reserved for individual benefit? These are difficult but important issues where context-specific understanding is needed in order to allay fears and communicate effectively to pre-empt a negative reaction. Focus group discussions with community leaders and selected representative groups within the population could be combined with key informant interviews or standardised questionnaires.

It is possible that low rates of testing are due partly to the perception that being diagnosed HIV-positive has only adverse effects, with no immediate benefit if treatment is not offered. The UTT strategy, incorporating the offer of immediate treatment, may overcome this disincentive to test and lead to higher uptake of VCT. This hypothesis needs to be evaluated.

Answers to the above questions would help inform the content of different modes of promotion of universal testing. Publicity using radio and newspapers and printed materials such as posters and leaflets are common methods used in health promotion [43,44]. When developing such publicity materials, data should be collected on understanding of and responses to different materials. During subsequent testing campaigns, data collected from those presenting for testing on their exposure and response to different promotion methods might guide the design of future campaigns. Patterns may emerge when data are matched with individual demographics (e.g. by age and sex) to analyse whether different promotion methods are effective in different population sub-groups.

A large scale multi-disease prevention package has been studied and shown to be highly effective in drawing large numbers of individuals at a community level to test by simultaneously offering VCT for HIV alongside provision of condoms, insecticide-treated bed nets and water filters [45] and optimises use of resources and opportunities. This integrated approach has the advantage of addressing multiple health promotion needs of a community and warrants further investigation in other settings.

### HIV Testing Methods

Most VCT services use rapid point-of-care tests (POCTs) for HIV infection, so that results can be provided rapidly to clients during the same visit [46]. The sensitivity, specificity and predictive values of these POCTs may need to be re-examined to determine whether these assays are reliable in the context of UTT. Limitations of POCTs have been identified [47-49] and, in the context of universal treatment, the concern is that ART may be initiated without confirmatory testing or assessment of CD4 count or other clinical parameters. The implications of false-positive results are therefore more serious than at present where immunological or clinical markers confirm immune suppression. Low specificity and positive predictive value of a POCT could lead to unnecessary treatment of HIV-negative individuals. Algorithms involving use of multiple POCTs, with laboratory confirmation for the small number of discordant or indeterminate results, may provide adequate performance but challenge resources.

Individuals with acute stage HIV infection, tested before they develop detectable antibody levels, will not be detected using current HIV POCTs. Although the point prevalence of such infections at the population level may be low, such individuals may contribute disproportionately to onward HIV transmission for both biological and behavioural reasons [50-57]. Hence, failure to detect and treat such individuals may significantly undermine the impact of UTT. The current methods to address this issue are limited. One option is to increase the frequency of HIV VCT (e.g. up to monthly), although this may not be practically feasible and may still miss a proportion of acute infections. This could be minimised by using laboratory-based fourth-generation HIV assays, but even with this technology a small number of highly infectious ‘acute cases’ will still be missed. Moreover, the use of these assays in African settings is called into question due to high rates of false positivity related to co-infection with other endemic organisms [58]. Currently under investigation is a combination antigen-antibody POCT [59] which has yet to be validated, but may miss fewer acute infections. Alternatively, HIV nucleic acid amplification testing (NAAT) of HIV antibody-negative patients presenting with symptoms consistent with acute HIV infection has been proposed [60].

### Frequency of Testing

Information is needed on the frequency of testing that should be recommended for a UTT programme. The only evidence concerning this specific issue is from mathematical models. Some mathematical models predict that annual HIV VCT is sufficient to confer a substantial reduction in HIV incidence in a high prevalence setting in sub-Saharan Africa [12,13,15-17]. Other models have shown that in populations with little variation in risk behaviour and random mixing, a 95% reduction in incidence could still be achieved if 80% of the population accepted HIV testing once every 3-4 years [14]. Further modelling, informed by empirical data on uptake of testing and re-testing, is needed to elucidate the choice of frequency of testing under a range of conditions.

Operationally the continued provision of frequent HIV VCT will be challenging and costly, and could potentially lead to population-level testing fatigue. The normalisation of HIV testing, opt-out testing and potential self-testing may be important strategies to investigate in the context of trials to deliver and evaluate frequent testing.

### Counselling Strategies

Existing models of counselling prior to an HIV test are generally not evidence-based [61] and there have been doubts as to whether pre-test counselling confers any benefit [62]. Current United States Centers for Disease Control and Prevention (CDC) guidelines only require informed consent for an HIV test, as for any other medical test, and pre-test counselling is not mandatory [63]. It is unclear whether VCT leads to the adoption of safer sexual behaviour, with conflicting findings from different studies [64,65]. A trial comparing two VCT delivery strategies in Zimbabwe found that HIV incidence was higher in the high-uptake VCT arm [30,66].

In the context of UTT, we need to examine whether counselling has an added role in changing attitudes to treatment - from the current understanding that treatment is for the very sick, to the concept of treatment irrespective of CD4 count or World Health Organization (WHO) stage. Different models of counselling could be compared in observational studies, and linkage with testing and subsequent uptake of treatment measured. Qualitative assessment of the added value of any such counselling would be informative both from user and provider perspectives.

As task-shifting options are considered, the acceptability - especially with respect to confidentiality - of using lay or community members to provide counselling must be examined and qualitative studies among non-users of services will be of particular importance.

Group pre-test counselling is used as a method to scale up VCT in many countries where low staffing levels struggle with the burden of clients attending for counselling and testing [67]. The capacity of group counselling to address pre-test issues in the context of UTT should be explored. Finally, where burden of workload is high, the impact on providers and how they cope warrant investigation.

### Uptake of Testing

Against the current backdrop of inadequate coverage and uptake, universal HIV testing is an ambitious goal. Routinely collected data from testing venues would provide the starting point to compare the reach of different models in terms of numbers testing and receiving their test results. More difficult will be ascertaining the proportion of a given population who have been tested. Accurate population information must be obtained and account will need to be taken of overlapping or ill-defined catchment areas of testing facilities, repeat testing at different facilities, and population migration and mobility.

Studying demographic associations with uptake of testing provided under different models of service provision would provide useful insights into which sub-groups of the population require further exploration and targeting, relating to gender, ethnicity, religion, socio-economic or educational characteristics.

Community-wide feasibility studies of pilot UTT programmes would provide a valuable opportunity to collect detailed data on uptake of testing, and to investigate reasons for uptake or non-uptake of testing using a combination of quantitative and qualitative methods. Such feasibility studies could also examine uptake of repeat testing and uptake of testing at different test sites, and could incorporate quantitative and qualitative studies of community and health-worker perceptions of the VCT programme.

### Costs

Cost-effectiveness studies of the various methods that may be piloted hold the key to identifying which strategies will be feasible for ministries of health and other providers who have to find sustainable ways to provide services with limited resources.

## UNIVERSAL HIV TREATMENT

### Introduction

Most countries in sub-Saharan Africa are struggling to maintain treatment initiation even for those meeting the current guidelines [68]. In the current environment, expanding access to ART for asymptomatic patients with higher CD4 counts, and offering immediate treatment to all HIV-positive individuals as required by the UTT strategy, will clearly be very challenging.

This section discusses some of the key issues and research questions that need to be addressed relating to linkages between testing and treatment, models of treatment provision, choice of treatment regimen, approaches to follow-up and monitoring, uptake and adherence, effects on other health services and costs. All such research will require effective community engagement and close collaboration with the existing health services. Key research questions and potential research methods are summarised in Table **2**.

### Linkages Between HIV Testing and Treatment

Current strategies to link those accepting VCT and found to be HIV-infected to services providing further clinical assessment and, if appropriate, initiation of ART often do not work effectively. Although this review focuses on research questions relating to UTT in Africa, data from other settings may be informative. An ongoing study in Washington, District of Columbia (DC) reported only 50% acceptance of referral to ART provision centres among those testing HIV-positive [69]. A national testing programme in South Africa reported successful testing of 1.7 million individuals between April and July 2010 but, of 300,000 HIV-positive subjects, only half were referred to any related health services and only an additional 3000 initiated ART during the first two months of the programme [70]. Barriers to accessing care include limited accessibility and coordination of services and lack of community engagement in programme planning [71]. Clearly, for a UTT programme to succeed in providing immediate ART for a large proportion of diagnosed HIV-positive patients in the community, challenges to linkage of services need to be addressed.

Physical proximity of testing and treatment sites, and convenient opening hours may address some of the barriers to linkage. In addition, assigning community health workers to individual patients to facilitate access to treatment, and trace those who test HIV-positive but do not present for treatment, could also facilitate linkage. An observational study has described the successful use of self-selected community ART groups to facilitate ART delivery and adherence^72^ and the extension of this concept to testing and linkage to care should be explored. However, the potential problems of lack of confidentiality and coercion, and consideration of non-participants, require close scrutiny in qualitative studies. In the absence of data supporting any one model of linkage to care, operational research is needed to evaluate such approaches.

### Models of Treatment Provision

Different approaches to treatment provision may be needed in different settings, and operational research and pilot studies will be needed to assess their feasibility and effectiveness. Some possible models are reviewed below but, to maximise coverage, a combination of approaches may be necessary.

#### Home-based care:

Whether or not VCT is provided through a door-to-door campaign, it may be possible to offer treatment and follow-up through a continuous system of home-based care. Such approaches have been shown to be effective and cost-effective in previous research in Uganda [73], although they usually incorporate some clinic visits. Advantages of this approach are that it shields newly diagnosed individuals from community scrutiny, does not require the construction of new facilities and may be more acceptable at a community level. Disadvantages include a high work-load for staff, disclosure within the home which may be associated with gender-based violence [74], and difficulties in accessing patients who are usually at work during the day unless evening visits are feasible.

#### Facility-based provision:

This is the standard approach at present but could be adapted to offer extended access to ART by increasing staff, clinic space or opening hours. An important issue is whether it is feasible to provide and monitor ART effectively at lower level health units than have traditionally provided ART, and this is related to the question of which cadres of staff are able to provide treatment. Integration of ART with other health services, for example those providing treatment for TB, maternal and child health care or family planning services, could also be considered for further exploration [75-77].

#### Community treatment centres:

If existing health facilities are not close enough to local communities, consideration could be given to establishing community-based treatment centres. These could either be permanent facilities or mobile clinics linked to locations such as markets, schools or religious venues, or could involve drug delivery at community level for stable patients, as an extension of facility-based provision [72].

#### Work-place provision:

This would probably be most appropriate at sites where there is a large workforce employed in a particular occupation, such as mines or plantations. Work-place VCT has been well accepted [30] and expansion of these facilities to ART provision would be a potentially effective method of delivering treatment.

Irrespective of the venue at which ART is provided, one of the key barriers to effective service provision is the shortage of trained health-care professionals to deliver therapy. While treatment of advanced stage HIV disease is often complicated by immune reconstitution inflammatory syndrome (IRIS), profound immunosuppression and opportunistic disease [78], provision of ART for asymptomatic individuals is likely to be less complex and might be delegated to less highly-qualified staff such as nurses or clinical officers. In a UTT programme, there will be a broad spectrum of HIV-positive patients requiring treatment, including both early and late stage infections. Task-shifting may be the only feasible approach given the large number of HIV-positive patients requiring treatment. Triage systems may be needed to ensure that those presenting with late-stage disease or with HIV-related illnesses are identified and treated by appropriately qualified staff and in suitable sites, while those who are well can be managed through mechanisms more adapted to lifestyle needs of healthy patients.

### Choice of Treatment Regimens

The choice of a safe, effective and acceptable first-line ART regimen that can be used irrespective of CD4 count in a UTT programme is critical in order to limit the potential development of drug resistance, drug-related toxicities and poor adherence. The regimen should be chosen to minimise pill burden and avoid need for refrigeration in order to increase adherence. The first-line regimen currently recommended by WHO [68] comprises tenofovir, emtricitabine and efavirenz or nevirapine. This would potentially be an appropriate choice for use in UTT, but clinical studies are needed to confirm this. Nevirapine-based regimens, whilst safe and well-tolerated for advanced stage disease with good safety data in pregnancy, confer risk of hepatic toxicity especially in women and in those with high CD4 counts (>350 cells/mm^3^). Although tenofovir/emtricitabine (or lamuvidine) and efavirenz may be the easiest, safest and most readily available option as recommended first-line therapy, the genital tract penetration of efavirenz is sub-optimal and could potentially lead to population-level development of transmitted drug-resistant variants. This latter aspect would require evaluation in UTT. Second-line therapy must also be available for those individuals unable to take first-line treatment due to toxicity, concomitant illness or drug resistance and treatment failure, and should adhere to national and WHO treatment guidelines.

Although there is emerging evidence of enhanced survival benefits with earlier ART [79,80], the optimal time to initiate ART for individual patient benefit remains uncertain [81-83]. While outcome is improved if ART is started in the presence of opportunistic infections and especially TB [84], how early to start ART in asymptomatic individuals is unknown [85]. This is currently under investigation in the *Strategic Timing of Antiretroviral Treatment (START)* trial, a randomised controlled trial (RCT) examining the effects of early initiation of ART (CD4 >500 cells/mm^3^) compared with current treatment guidelines (CD4 <350 cells/mm^3^) which will report in 2015 [86]. The use of ART outside current national and WHO treatment guidelines will require careful discussion with local stakeholders and national authorities.

Any ART agent may be associated with drug-related toxicities [87], for example tenofovir with renal impairment [88] and zidovudine with anaemia [89]. The *2NN* study reported an association of nevirapine with an increased frequency of serious adverse events among patients with high CD4 counts [90]*. *In addition, although there have been concerns over the potential teratogenic effects associated with efavirenz [91,92], a more recent meta-analysis has found no increased risk of birth defects among women exposed to efavirenz during the first trimester of pregnancy compared with other ART agents [93]. A reasonable strategy to test the UTT approach would be to adopt one of the currently recommended first-line therapy regimens, avoiding the use of stavudine wherever possible. Clearly, this may pose feasibility concerns in resource-limited settings which are struggling to progress to the recently recommended first-line regimen, despite the availability of generic once daily formulations and recent price reductions [94]. Clinical research is needed to evaluate the acceptability, tolerability and cost-effectiveness of alternative first- and second-line regimens when delivered in a UTT programme to asymptomatic patients with high CD4 counts as well as patients with more advanced immunosuppression. Data can be obtained from observational studies and potentially from RCTs.

### Approaches to Monitoring and Follow-Up

Routine inclusion of laboratory-based testing involves additional logistical, resource and staff constraints, potentially undermining the feasibility of delivering an effective UTT programme. Depending on the disease stage of the individual patient and choice of ART regimen, toxicity monitoring should be kept to the minimum required to avoid increased risk to the patient. The *Development of Antiretroviral Therapy in Africa (DART)* trial established the safety and long-term sustainability of ART provision without laboratory-based monitoring in rural Uganda and Zimbabwe, although after 2 years there was a slight difference in mortality between the two arms of the trial [81,95]. Many patients on immediate treatment in a UTT model will be at an earlier stage of HIV disease progression than those in the *DART* trial, but there is no reason to believe that they will have greater monitoring needs, and the *DART* findings remain relevant for UTT.

The level of monitoring required will vary according to treatment regimen, but if standard first-line therapy is employed this could be minimal. Renal toxicities could be monitored using urine protein/albumin levels tested by dipstick as an initial screening procedure. Only those with significant proteinuria would then be referred for blood testing. Management of any complications would be according to national guidelines and would necessitate full laboratory tests as indicated. It will not be feasible when developing the UTT concept to regularly monitor CD4 counts or viral load using current laboratory-based testing. The introduction of POCTs for CD4 count [96] may make this more feasible, although these tests will require careful field evaluation.

The adequacy of monitoring and follow-up will need careful assessment in feasibility studies and RCTs of UTT interventions. For example, a random sample of patients in such studies could be monitored more frequently or using additional laboratory tests (e.g. renal and liver function, blood cell counts) and the data used to assess the potential adequacy of less intensive follow-up schedules.

### Adherence to ART

The risk of developing viral resistance to ART is directly linked to adherence to therapy [97] and drug choice, with enhanced viral suppression conferred by boosted protease inhibitor- or non-nucleoside reverse transcriptase inhibitor-containing regimens compared with triple nucleoside reverse transcriptase inhibitor combinations [98]. Stopping ART due to concomitant ill health, drug supply problems, ambivalence to continuing therapy or cultural and health beliefs confers not only increased risks of adverse events [99-101] but additional problems associated with viral rebound leading to enhanced risk of onward transmission or the development of drug resistance [102,103].

Adherence may be reduced in a UTT programme, since many of the patients started on ART may have had no clinical symptoms associated with their HIV infection. Patient messaging will need to be adapted taking this new treatment paradigm into account. Levels of adherence will need careful assessment in feasibility studies and RCTs of UTT interventions. Data from such studies can be used in mathematical modelling to predict the likely long-term effects of sub-optimal adherence on drug resistance and HIV transmission.

### Uptake of Treatment

Uptake of immediate ART can be expected to vary in different settings [69,104] and feasibility studies are needed to measure the actual uptake achieved by different UTT approaches in different areas in sub-Saharan Africa. Such studies will require careful definition of the denominator population of individuals who test positive through any of the testing services provided, paying careful attention to the need to avoid double counting if individuals test at more than one centre. A definition will also be needed for “immediate” uptake of treatment, which might for example be taken as 3 months from the time of HIV diagnosis.

By selecting random samples of those accepting and declining immediate treatment, and using a mix of quantitative and qualitative methods, the same studies could investigate reasons for not taking up the offer of immediate ART. The findings of such studies could be used to further improve the services to overcome barriers to uptake of treatment.

### Outcome of Treatment

Feasibility studies of UTT programmes will also need to collect detailed data on the outcomes of treatment. These may include retention under follow-up, adherence to treatment, adverse effects, viral suppression and the development of drug resistance. Some of these outcomes could be measured through more intensive observations on a random sample of patients starting on treatment. Case-control studies could be performed on patients with good and poor outcomes to investigate risk factors for poor outcomes. As for testing services, qualitative studies could provide valuable information on patient and health-worker perceptions of the treatment programme and how it could be improved.

### Effects of UTT on Health Services

There are concerns that substantial increases in numbers of patients on ART could lead to over-burdening of poorly-resourced and understaffed health facilities, leading to adverse effects on health services. Conversely, effective ART provision for a high proportion of HIV-positive individuals in a community should lead to substantial decreases in HIV-related morbidity, and this should reduce the incidence of opportunistic infections requiring diagnosis and treatment. A study in Zambia showed ancillary benefit to routine clinical services when disease-specific research was combined with service delivery [105]. Observational studies, for example during feasibility studies or RCTs, should be carried out to collect detailed data on clinic burden and on the delivery of other routine health services.

It has been suggested that the efficiency of service delivery could be increased if ART delivery could be integrated with the provision of treatment and care for other chronic conditions, including TB and non-communicable diseases such as diabetes and hypertension, which are increasing in incidence in many parts of sub-Saharan Africa. Operational research is needed to explore such treatment synergies.

### Costs

Policy decisions on implementation of the UTT strategy will depend critically on reliable cost-effectiveness estimates, particularly since initial costs of UTT roll-out may be very high. Detailed costing studies must be incorporated in feasibility studies and other operational research studies of UTT to provide the basis for such cost-effectiveness estimates. Drug costs comprise only a small part of the overall cost of delivering immediate ART, and account needs to be taken of other costs including personnel, laboratory testing, training, supervision, monitoring and the costs of referral and second-line treatment.

## EFFECTS OF UTT ON HIV TRANSMISSION

### Introduction

Previous sections have discussed operational research relating to the provision of services to deliver the UTT strategy. If the strategy is shown to be feasible and acceptable in resource-poor settings in sub-Saharan Africa, the next question is what impact this would have on HIV infection. In this section, we discuss the research that will be needed to determine the effect of UTT on HIV transmission both at individual and population levels. We also consider studies to examine the cost-effectiveness and adverse effects of the intervention. Key research questions and potential research methods are summarised in Table **3**.

### Effects of ART on HIV Transmissibility at Individual Level

Model projections of the impact of UTT on HIV transmission depend on assumptions regarding the effect of ART on HIV transmissibility from an HIV-infected individual to his or her sexual partners. HIV plasma viral load is known to be a key determinant of HIV transmissibility [106,107] and ART, if delivered effectively, reduces plasma viral load to an undetectable level in a high proportion of patients [108]. It is therefore assumed that ART will reduce the risk of HIV transmission to a very low level. For accurate modelling, however, more precise data are needed on effects of ART on transmissibility. These data are also needed to give clear and accurate information to HIV-positive patients on ART concerning the risk of transmitting HIV to their sexual partners, and this is clearly of special concern in HIV-discordant long-term partnerships.

The early termination and results of the *HPTN 052* trial have been described earlier [23]. As initiation of ART at higher CD4 counts is considered, further research will be needed to measure the effects of immediate *versus *deferred treatment on both HIV transmission and HIV-related morbidity and disease progression.

Trials looking directly at transmission by following up HIV-discordant partners are logistically challenging, and need to be large since transmission rates are often reduced to low levels because the HIV status of the partnership is known and intensive prevention counselling is delivered for ethical reasons. Despite limitations related to selection effects and confounding, it is therefore likely that most data will continue to come from observational studies in which HIV incidence in HIV-discordant couples is examined in relation to the ART treatment status of the HIV-positive index case. In a recently published landmark study, an observational analysis was carried out among 3381 HIV-discordant couples taking part in a multi-centre RCT of *Herpes* suppressive therapy for HIV prevention [109]. In these couples, the HIV-positive index partner initially had a CD4 count of 250 cells/mm^3 ^or over and did not meet national guidelines for ART initiation. Subsequently, 349 index cases commenced ART and the adjusted rate ratio for HIV infection in their sexual partners compared with those not on ART was 0.08, indicating a 92% reduction in the risk of HIV transmission, and only one genetically-linked HIV transmission was observed among those on ART. However, the confidence interval for the rate ratio was very wide (0.00 - 0.57) and further data are needed to obtain more precise estimates. As well as further observational analyses based on current and future cohorts of patients on and off treatment, there will be a place for further systematic reviews bringing together all available data on this question.

Genital shedding of HIV is known to be increased in the presence of other sexually transmitted infections (STIs), which are highly prevalent in some parts of sub-Saharan Africa. Even though ART reduces plasma viral load to low levels, there is a concern that spikes of HIV genital shedding may occur during episodes of STIs, possibly increasing the risk of HIV transmission [110-112]. Further data are needed from studies of genital shedding of HIV in index cases with different CD4 counts, plasma viral load and ART treatment status, and with or without concomitant STIs. Future observational analyses of HIV transmission to sexual partners should also collect prospective data on the incidence of STIs.

### Effects of UTT on HIV Transmission at Population Level

The rationale underpinning the UTT strategy is that a large proportion of HIV transmission events take place in index cases who are not on ART because they have not yet been diagnosed HIV-positive, have been diagnosed but are not yet eligible for ART, or are eligible for ART but have not yet started on ART. The impact of UTT on HIV transmission will depend partly on the size of this untreated proportion and on the extent to which UTT can reduce it. However, the overall impact on HIV incidence at population-level will also reflect *indirect effects* of the intervention, since the new cases of HIV infection averted will lead to a further reduction in index cases and hence future transmission events. Prediction of population-level impact under different conditions is complex and is likely to depend on mathematical modelling which needs to be based on realistic assumptions and input parameters. However, proof of concept is likely to come from a cluster-randomised trial of the UTT strategy and this is discussed below.

Initial work is needed to address the question of what proportions of new HIV infections in different populations are attributable to index cases at different CD4 counts and stages of HIV progression. This is likely to involve a combination of data collection and mathematical modelling. Data are needed on uptake of HIV testing, delay before new HIV infections are diagnosed, coverage of ART, delay before onset of ART and the CD4 count at which ART commences. All these parameters are likely to vary substantially between countries and settings. Together with the available data from observational studies on the association between stage of HIV progression and HIV transmissibility, these data can be used in mathematical models to estimate the proportion of HIV transmission events occurring at different CD4 counts or stages of HIV progression.

Based on this initial work, the mathematical models can then be used to explore the likely effects on HIV transmission of a UTT intervention. Scenario analysis will be needed to examine the effect of a range of assumptions about the performance of UTT, including uptake of testing, acceptance of immediate treatment and adherence to ART [113]. Relatively simple models can be used to make rough estimates of short-term effects on transmission based only on changes in the treatment status of index cases and consequent changes in HIV transmissibility. Long-term projections of population-level impact, however, will require the application of more sophisticated HIV transmission models that take account of sexual networks and the dynamics of transmission in the population as well as capturing the effects of behavioural disinhibition and indirect “herd” effects resulting from reductions in the number of new index cases.

The feasibility studies discussed in earlier sections of this paper would help to provide the data to guide parameter choice for future evidence-based modelling work. Where data are unavailable, a range of plausible assumptions can be explored. Modelling can also be done to investigate the effects of different aspects of UTT implementation, such as the proposed frequency of HIV testing and re-testing in the community as well as levels of coverage and uptake.

Implementation of UTT will depend not only on evidence of *population-level effectiveness* but also on *affordability* and *cost-effectiveness*. Proposed feasibility studies of UTT interventions should collect detailed data on costs of implementation and the components of these costs. These data can be used by economists to estimate the likely costs of UTT interventions in a range of settings with different input prices. Data on estimated costs can be integrated with the mathematical models to obtain estimates of cost-effectiveness under different conditions, which may be expressed for example as cost per HIV infection averted or cost per disability-adjusted life year (DALY). These estimates will be based on differences in projected numbers of HIV infections over a defined study period (for example 10 or 15 years) for the proposed UTT intervention and the current level of diagnosis and treatment, combined with data on differences in costs under the two scenarios. When computing costs, it is important that account is taken not only of the direct costs of implementing the UTT intervention but also of *costs averted* due to future reductions in HIV infections needing care and other services as well as reductions in HIV-related morbidity due to earlier treatment and higher uptake of treatment. Previous modelling work suggests that the saving of hospital and other health-care costs for these illnesses will largely offset the costs of implementing UTT at least in a South African setting [12,21,114]. It may also be helpful to model the cost-effectiveness of UTT compared with that of a more limited intervention aimed at expanding testing and increasing the numbers of patients treated promptly according to current treatment guidelines.

While modelling should provide useful projections of cost-effectiveness under a range of conditions, there will always be uncertainties about the validity of such projections given the large number of assumptions required, many not well supported by empirical evidence. UTT represents a controversial and logistically very challenging intervention, with a range of possible adverse effects, ranging from increased stigma and behavioural disinhibition to increases in drug resistance and over-burdening of health services. A decision to go ahead with wide-scale implementation is likely to depend on proof of concept obtained through one or more rigorously conducted cluster-randomised trials. Such trials could directly collect data on population-level impact on HIV incidence as well as on implementation costs and many other outcomes including the potential adverse effects noted above.

## CLUSTER-RANDOMISED TRIALS OF UTT: DESIGN CONSIDERATIONS

### Introduction

We have argued that rigorous proof of concept of UTT as an HIV prevention strategy is likely to depend on evidence from a *cluster-randomised trial *(CRT) of the impact of UTT at population level. In this section, we briefly discuss key design aspects of such a trial, which is likely to involve random allocation of a number of study communities to the UTT intervention or a standard of care control arm, and follow-up over several years to record effects on HIV incidence at population level.

### Rationale for CRT Design

RCTs remain the gold standard for the evaluation of health interventions, where they are feasible. Cluster randomisation is called for where the intervention has by its nature to be implemented at population level, as is the case for UTT. Moreover, cluster randomisation allows the total effect of the intervention, including indirect “herd” effects, to be captured [115,116]. By comparison of both effects and costs between intervention and control clusters, a CRT can provide rigorous empirical estimates of effectiveness and cost-effectiveness to guide the decisions of policy-makers regarding the implementation of health interventions.

### Study Populations and Definition of Clusters

It is likely that any CRT of the UTT strategy will be a multi-site trial involving sites in several countries in sub-Saharan Africa, for two reasons. First, a multi-country study will help to ensure that the results of the trial are generalisable and reflect the performance of the strategy under a range of conditions. Second, the size of such a trial and the large number of clusters required are likely to call for a multi-country study.

It is likely that the effects of the intervention will show some variation due to differences in the existing coverage of testing and treatment services, variations in the uptake and delivery of the UTT strategy, underlying differences in the epidemiology and transmission dynamics of HIV and other contextual factors. It is important to note that the proposed CRT will be powered to determine the overall effect of UTT, averaged over sites. While the data may give some indication of relative effects of UTT in different settings, the study will not be powered to provide precise data on variations in effectiveness between countries. Once again, there will be a role for mathematical modelling in taking the empirical data from the CRT and using them to provide refined estimates of effectiveness and cost-effectiveness in different settings.

The choice of clusters is likely to depend on the level at which the intervention is implemented, as well as the transmission dynamics of HIV infection. Ideally, to correctly capture the indirect effects of the intervention and to avoid contamination due to sexual contacts with individuals from outside the cluster, the cluster would be large enough to ensure that most sexual contacts occur within the cluster. However, the requirement for a relatively large number of clusters and the need to constrain costs will necessitate a compromise between feasibility and validity. In practice, the cluster might be defined as the catchment population of a health facility through which the UTT intervention is to be delivered and coordinated. To minimise contamination, and problems due to overlapping catchments of different health facilities, it may be preferable to select widely dispersed rural communities or small towns, rather than areas within large urban conurbations.

### Standard of Care in Control Arm

A critical component of the CRT design is comparison of effects in intervention clusters with comparable clusters in a control arm. An important issue for CRTs of UTT is what services will be provided in the control arm. To maximise study power and to provide the most relevant evidence for health policy, it would arguably be desirable for the control arm to receive the current level of service provision in the study community with no additional interventions. However, the ethical acceptability of enrolling and following up study communities where currently recommended criteria for service provision are not adhered to, as is likely to be the case in many resource-poor settings, would need to be carefully considered. International guidelines on this issue have been an area of intense debate and disagreement, and are under constant review. Responsibilities to trial participants have to be balanced against the urgent need for valid data to guide national and international policy, as well as concerns over providing enhanced service levels which are unlikely to be sustained after the end of a study.

One option would be to enhance service provision to an acceptable “standard of care”, ensuring that anyone wishing to be tested for HIV has easy and convenient access to a VCT service of acceptable quality, and that all those diagnosed HIV-positive have access to ART according to current national treatment guidelines. This would certainly result in higher ART coverage than at present, thus diluting any difference in outcomes between the study arms, and this would need to be allowed for in sample size calculations. An alternative is to provide communities in the control arm with some other intervention, unrelated to HIV infection, that would be of intrinsic value to the community.

It is likely that random samples of adults from the study communities will be followed up to measure effects on HIV incidence. Enrolling individuals to a research cohort of this kind may impose additional constraints regarding the responsibilities of the research team. What access would this cohort have to HIV counselling, testing and treatment? Would there be an expectation that proven HIV prevention methods (such as male circumcision) should be made available to these cohorts? These are difficult questions that would require careful consideration and discussion with national authorities and ethical review committees.

### Duration of Follow-Up

It is likely that the UTT strategy will take several years to show its full effect for two main reasons. First, it will take some time to introduce the intervention into the study communities and to expand coverage so that a maximal proportion of the population have been tested and a high proportion of those diagnosed HIV-positive have been started on ART. Second, indirect “herd” effects of the intervention will accumulate over time, and a long enough follow-up period is needed for these to be captured. Similarly, it may also take some time for potential adverse effects of the intervention, such as HIV-related stigma or behavioural disinhibition, to be observable. Conversely, some initial problems with the intervention may be resolved over time; for example, it may take time to establish the trust of the community in the confidentiality and quality of the services, and uptake may increase substantially once this has been achieved.

Previous mathematical modelling showed that UTT would have an immediate effect on HIV incidence but that effects would accumulate over time. “Elimination” of HIV as a public health problem was only projected after 15-20 years. For these reasons, a CRT would require a long enough follow-up period so that at least the early effects of the intervention are detectable, and this is likely to be at least two to three years.

### Primary and Secondary Endpoints

The primary objective of a CRT would be to measure the impact of the UTT intervention on HIV incidence at population level, and so HIV infection in the general population of the study communities would be the primary endpoint. If a reliable assay that can detect recent HIV infection is available and validated, then HIV incidence could be measured through a cross-sectional survey of a random sample of the population at the end of the follow-up period. Unfortunately, none of the existing assays have been validated in the likely study populations for a CRT, where HIV subtypes differ from those that predominate in settings where most of these assays have been developed. If such assays are not available, measurement of HIV incidence is likely to require enrolment and follow-up of a cohort which might be a random sample of the adult population. Cohort follow-up brings a number of problems, including difficulties in ensuring a high rate of follow-up, particularly when mobility and migration rates are high, and “cohort attrition” leading to declining incidence over time.

There are likely to be numerous secondary endpoints and these are likely to include HIV-related morbidity and mortality; uptake of testing; uptake of treatment; adherence and treatment failure; drug resistance and toxicity; HIV-related stigma; TB incidence; HIV vertical transmission; and behavioural disinhibition.

### Sample Size

Special methods are required for sample size determination for a CRT [116]. These depend on the expected incidence of HIV; the effect size on which the study is to be powered; and the between-cluster coefficient of variation in the primary endpoint. Both the number of clusters per study arm and the number of individuals to be followed up in each cluster need to be specified. Note that the latter may be a random sample of the community, and it is not necessary to follow up the entire population of each community.

To illustrate the likely size of a CRT, suppose HIV incidence in adults is approximately 1 per 100 person-years in control communities, and we wish to detect a 40% reduction in HIV incidence with 90% power, and the coefficient of variation is 0.25 indicating that HIV incidence varies roughly between 0.5 and 1.5 per 100 person-years in the control arm. Then if we select a random sample of 1000 adults in each cluster and follow them up for 3 years giving 2550 person-years of observation per cluster (assuming 15% loss of person-years), we would need 11 clusters per arm (a total of 22 clusters) with an unmatched design. Table **4** shows sample size requirements for a CRT under a range of assumptions.

## CONCLUSIONS

In this paper, we have reviewed the research questions that need to be addressed to provide the evidence needed to inform policy on the implementation of the UTT strategy, and outlined possible research methods that could be used to answer these. Some of these studies can be done using existing data, but new field research will also be needed.

In particular, we propose a phased sequence of studies, commencing with pilot studies looking at the feasibility and acceptability of the intervention. If these show favourable results, it might then be appropriate to proceed to a full-scale CRT to measure the impact of the intervention on HIV incidence at population level, as well as a wide range of secondary outcomes. Such a trial would produce rigorous evidence on the beneficial and adverse effects of the UTT intervention which, together with cost data, would provide estimates of cost-effectiveness to guide policy decisions on the potential wide-scale implementation of the intervention.

One of the key limitations of the proposed CRT is that it is unlikely to be feasible to follow up the study communities for more than a few years, whereas model projections show that the full impact of such an approach may not be seen for 15 to 20 years. However, these projections do predict a substantial early effect of the intervention, and observations confirming such an effect would provide strong support for wider introduction. Mathematical models could be fitted to data from a CRT on early effects of UTT and used to refine estimates of longer-term impact. In any case, the need for more effective prevention strategies is urgent, and cannot wait for the results of a trial extending for 10 years or more.

It is by no means certain that initial feasibility studies will show the UTT approach to be feasible or acceptable in resource-poor settings. More limited and targeted approaches to intensified delivery of HIV testing and treatment, while not resulting in “elimination”, may nevertheless have an appreciable effect on HIV transmission. We suggest that work should continue on such approaches in parallel to the proposed initial studies of UTT.

The concept of UTT fits into the broader paradigm of *combination HIV prevention*. There is increasing recognition that single interventions are unlikely to be sufficient to bring the HIV epidemic under effective control in the most severely affected countries, and that a combination of partially effective methods will be required [117]. UTT itself represents a combination prevention tool as it involves a combination of testing, linkage to care and early treatment. Achieving high uptake of testing through a UTT programme provides an opportunity to offer other proven interventions such as male circumcision, condoms and potentially microbicides or oral PrEP. The CRT design discussed above could be extended to measure the population-level impact of a combination prevention package that includes UTT combined with other interventions.

Whether or not the research studies outlined in this paper show that UTT is a feasible and effective HIV prevention intervention, they will provide a wide range of data that will be of value in guiding the strengthening of services for testing and treatment in sub-Saharan Africa - for both individual and public health benefit.

## Figures and Tables

**Table 1.** Key Research Questions Related to Counselling and Testing in Universal Testing and Treatment (UTT) and Suggested Research Methods

Key Research Areas	Questions to be Answered	Randomised Controlled trials	Observational Studies	Qualitative/Mixed Methods Research	Modelling	Cost Studies
**Models of service provision**	Where should VCT services be provided? Who should deliver VCT? What is the added role of self-testing? What is the role of expanded PIT in medical settings?	√	√	(√)		
**Promotion of universal testing**	What beliefs and perceptions need to be understood to appropriately target promotion of UTT in a given community?		√	√		
	Which subsets of the population are captured by different modes of promotion? What modes of promotion are most effective in achieving uptake of testing?	√	√	√		
**Test methods**	What is the predictive value of POCT in the context of UTT for a given population prevalence of HIV?		√			
**Frequency of testing**	What frequency of testing (and treatment) is needed to reduce the R0 of HIV to <1?				√	
**Counselling strategies**	What is the evidence on the effectiveness of pre-test counselling approaches?	√	√	√		
	How does post-test counselling affect behaviour change, HIV incidence and repeat testing?	√	√			
	How does group pre-test with individual post-test counselling compare with individual pre and post-test counselling?	√	√	√		
**Uptake of testing**	What are the barriers to testing? What aspects of service provision ameliorate identified barriers?			√		
	What proportion of a given community has participated in VCT and repeat testing? What factors are associated with non-uptake and failure to re-test?		√			
**Costs**	What are the training, staffing, equipment and site costs?		√		√	√

UTT, Universal Testing and Treatment; VCT, Voluntary Counselling and Testing; PIT, Provider-Initiated Testing; POCT, Point-of-care Testin

**Table 2.** Key Questions Related to Treatment in Universal Testing and Treatment (UTT) and Suggested Research Methods

Key Research Areas	Questions to be Answered	Randomised Controlled Trials	Observational Studies	Qualitative /Mixed Methods Research	Modelling	Cost Studies
**Linkages between testing and treatment**	How well does a given VCT model achieve linkage with treatment? What factors act as barriers to uptake of treatment after testing positive? What programmatic factors are associated with better linkage between testing and treatment initiation?	√	√	√		
**Models of treatment provision**	Which models of treatment provision are most effective and acceptable? Where should treatment be provided? Who should deliver these treatment services?	√	√	√		
**Choice of treatment regimens**	Which treatment regimens are safe, effective and acceptable to use as first-line treatment?	√	√	(√)		
	Which treatment regimens are feasible and sustainable for use in resource-limited settings?	√	√	√		
	Which treatment regimens are associated with ART failure and drug resistance at different time points?	√	√			
**Monitoring and follow-up **	What are the monitoring requirements of a given treatment regimen?	√	√			
	Are there different monitoring requirements for patients who were sick when they started treatment compared with those who were well?	√	√	√		
	What are the evolving challenges in maintaining follow-up over time?		√	√		
**Adherence to ART**	What patient factors are associated with good adherence (>90%) at different time points on treatment?		√	√		
	What programmatic factors are associated with good adherence at different time points on treatment?		√	√		
**Uptake of treatment**	What proportion of HIV-infected individuals in a population are on treatment at different time points?		√			
	What is the impact of a given proportion on treatment on HIV transmission?				√	
**Effects of UTT on health services**	What is the impact of UTT on health services in a given location?		√	√	√	√
	What are the cost implications (financial and human resources) of UTT over time, including costs averted?			√	√	√

**Table 3.** Key Questions Related to Population-Level Effects of Universal Testing and Treatment (UTT) and Suggested Research Methods

Key Research Areas	Questions to be Answered	Randomised Controlled Trials	Observational Studies	Qualitative/Mixed Methods Research	Modelling	Cost Studies
**Effect of UTT on HIV transmissibility at individual level**	What are the effects of ART on genital shedding of HIV? What are the effects of ART and different levels of genital HIV shedding on risk of transmission to sexual partners? What are the effects of ART on HIV transmissibility in the presence of concomitant sexually transmitted infections?	√	√		(√)	
**Effects of UTT on HIV transmission at population level**	What proportions of new HIV infections are attributable to index cases at different CD4 counts and HIV clinical stages?		√		√	
	What is the impact of UTT on population-level HIV incidence at different time points? How does the impact of UTT on HIV incidence depend on key process variables such as uptake of testing and treatment, adherence and retention?	√	(√)		√	
**Other effects of UTT**	What are the effects of UTT on HIV-related morbidity and mortality? What are the effects of UTT on the incidence and prevalence of TB and other opportunistic infections? What are the effects of UTT on vertical transmission of HIV? What are the effects of UTT on the incidence of treatment failure, toxicity and drug resistance? Is delivery of UTT associated with behavioural disinhibition?	√	(√)	√	√	
**Cost-effectiveness of UTT for HIV prevention**	What are the total *per capita* costs of implementing a UTT programme at population level? What cost savings are provided by reductions in HIV incidence and HIV-related morbidity? What is the overall cost-effectiveness of UTT expressed per HIV infection averted or per DALY? How do the effectiveness and cost-effectiveness of UTT compare with those of more limited treatment programmes?	√	(√)		√	√

UTT, Universal Testing and Treatment; ART, Antiretroviral Therapy; TB, Tuberculosis; DALY, Disability-adjusted Life Year.

**Table 4.** Sample Size Requirements for Cluster Randomised Trial of Universal Testing and Treatment (UTT) for HIV Prevention. Table Shows Number of Clusters Required in Each Study Arm Under a Range of Scenarios

HIV Incidence in Control Arm (/100py)	Sample Size of Cohort Per Cluster *	Coefficient of Variation	Impact on HIV Incidence (1 - RR) x 100%	Power	Clusters Required Per Study Arm

1.0 **	**1000**	**0.25**	**40%**	**90%**	****11****

**0.5**	1000	0.25	40%	90%	**15**
**1.0**	1000	0.25	40%	90%	**11**
**1.5**	1000	0.25	40%	90%	**10**
**2.0**	1000	0.25	40%	90%	**9**

1.0	**500**	0.25	40%	90%	**15**
1.0	**1000**	0.25	40%	90%	**11**
1.0	**2000**	0.25	40%	90%	**9**

1.0	1000	**0.20**	40%	90%	**9**
1.0	1000	**0.25**	40%	90%	**11**
1.0	1000	**0.30**	40%	90%	**14**

1.0	1000	0.25	**30%**	90%	**20**
1.0	1000	0.25	**40%**	90%	**11**
1.0	1000	0.25	**50%**	90%	**7**

1.0	1000	0.25	40%	**80%**	**9**
1.0	1000	0.25	40%	**90%**	**11**

* Assumes cohort is followed up for 3 years with 15% loss of person-years of follow-up.

** Top row shows default scenario discussed in text. Suppose HIV incidence in adults is approximately 1 per 100 person-years in control communities, we wish to detect a 40% reduction in HIV incidence with 90% power and the coefficient of variation is 0.25 indicating that HIV incidence varies roughly between 0.5 and 1.5 per 100 person-years in the control arm. Then if we select a random sample of 1000 adults in each cluster and follow them up for 3 years giving 2550 person-years of observation per cluster (assuming 15% loss of person-years), we would need 11 clusters per arm (a total of 22 clusters) with an unmatched design.

UTT, Universal Testing and Treatment; py, person-years; RR, relative risk.

## Footnotes

* While this paper was in press, two RCTs (Partners PrEP and TDF-2) have found that the use of oral daily PrEP confers a significant reduction in HIV acquisition in heterosexual populations ^1,2^.

## ABBREVIATIONS

AIDS: = Acquired Immune Deficiency Syndrome
ART: = Antiretroviral Therapy
CAPRISA: = Centre for the AIDS Programme of Research in South Africa
CDC: = (United States) Centers for Disease Control and Prevention
CRT: = Cluster-Randomised Trial
DALY: = Disability-Adjusted Life Year
DART: = Development of Antiretroviral Therapy in Africa
DC: = District of Columbia
HAART: = Highly Active Antiretroviral Therapy
HIV: = Human Immunodeficiency Virus
HPTN: = HIV Prevention Trials Network
iPrEx: = Pre-exposure Prophylaxis Initiative
IRIS: = Immune Reconstitution Inflammatory Syndrome
MRC: = Medical Research Council
NAAT: = Nucleic Acid Amplification Test
PIT: = Patient-Initiated Testing
POCT: = Point-Of-Care Test
PrEP: = Pre-exposure Prophylaxis
Py: = person-years
R_0_: = Reproductive Rate
RCT: = Randomised Controlled Trial
RR: = Relative Risk
START: = Strategic Timing of Antiretroviral Treatment
STI: = Sexually Transmitted Infection
TB: = Tuberculosis
UK: = United Kingdom
UTT: = Universal Testing and Treatment
VCT: = Voluntary Counselling and Testing
WHO: = World Health Organization

## References

[1] World Health Organization, UNAIDS Towards universal access:
scaling up priority HIV/AIDS interventions in the health sector.
WHO 2009 Progress Report. Available at: http://www.who.int/hiv/pub/2009progressreport/en/index.html.

[2] Padian NS, McCoy SI, Balkus JE, Wasserheit JN (2010). Weighing the gold in the gold standard: challenges in HIV prevention research. AIDS.

[3] Gray RH, Kigozi G, Serwadda D (2007). Male circumcision for HIV prevention in men in Rakai, Uganda: a randomised trial. Lancet.

[4] Bailey RC, Moses S, Parker CB (2007). Male circumcision for HIV prevention in young men in Kisumu, Kenya: a randomised controlled trial. Lancet.

[5] Auvert B, Taljaard D, Lagarde E, Sobngwi-Tambekou J, Sitta R, Puren A (2005). Randomized, controlled intervention trial of male circumcision for reduction of HIV infection risk: the ANRS 1265 Trial. PLoS Med.

[6] Grosskurth H, Mosha F, Todd J (1995). Impact of improved treatment of sexually transmitted diseases on HIV infection in rural Tanzania: randomised controlled trial. Lancet.

[7] Rerks-Ngarm S, Pitisuttithum P, Nitayaphan S (2009). Vaccination with ALVAC and AIDSVAX to prevent HIV-1 infection in Thailand. N Engl J Med.

[8] Mayaud P, Legoff J, Weiss HA (2009). Impact of acyclovir on genital and plasma HIV-1 RNA, genital herpes simplex virus type 2 DNA, and ulcer healing among HIV-1-infected African women with herpes ulcers: a randomized placebo-controlled trial. J Infect Dis.

[9] Abdool Karim Q, Abdool Karim SS, Frohlich JA (2010). Effectiveness and safety of tenofovir gel, an antiretroviral microbicide, for the prevention of HIV infection in women. Science.

[10] Grant RM, Lama JR, Anderson PL (2010). Preexposure chemoprophylaxis for HIV prevention in men who have sex with men. N Engl J Med.

[11] FEM-PreP Project: Study to assess the role of Truvada in
preventing HIV acquisition in women. FHI 360 2011. Avaiable at: http://www.fhi.org/en/Research/Projects/FEM-PrEP.html.

[12] Granich RM, Gilks CF, Dye C, De Cock KM, Williams BG (2009). Universal voluntary HIV testing with immediate antiretroviral therapy as a strategy for elimination of HIV transmission: a mathematical model. Lancet.

[13] Dieffenbach CW, Fauci AS (2009). Universal voluntary testing and treatment for prevention of HIV transmission. JAMA.

[14] Dodd PJ, Garnett GP, Hallett TB (2010). Examining the promise of HIV elimination by 'test and treat' in hyperendemic settings. AIDS.

[15] Garnett GP, Baggaley RF (2009). Treating our way out of the HIV pandemic: could we would we should we?. Lancet.

[16] Montaner JS, Hogg R, Wood E (2006). The case for expanding access to highly active antiretroviral therapy to curb the growth of the HIV epidemic. Lancet.

[17] Dias Lima V, Johnston K, Hogg RS (2008). Expanded access to highly active antiretroviral therapy: a potentially powerful strategy to curb the growth of the HIV epidemic. J Infect Dis.

[18] Blower S, Ma L, Farmer P, Koenig S (2003). Predicting the impact of antiretrovirals in resource-poor settings: preventing HIV infections whilst controlling drug resistance. Curr Drug Targets Infect Disord.

[19] Baggaley RF, Fraser C (2010). Modelling sexual transmission of HIV: testing the assumptions, validating the predictions. Curr Opin HIV AIDS.

[20] Zachariah R, Harries AD, Philips M (2010). Antiretroviral therapy for HIV prevention: many concerns and challenges, but are there ways forward in sub-Saharan Africa?. Trans R Soc Trop Med Hyg.

[21] Wagner B, Blower S (2010). Costs of eliminating HIV in South Africa have been underestimated. Lancet.

[22] Wagner BG, Kahn JS, Blower S (2010). Should we try to eliminate HIV epidemics by using a 'Test and Treat' strategy?. AIDS.

[23] Cohen MS, Chen YQ, McCauley M (2011). Prevention of HIV-1 infection with early antiretroviral therapy. N Engl J Med.

[24] De Cock KM, Bunnell R, Mermin J (2006). Unfinished business--expanding HIV testing in developing countries. N Engl J Med.

[25] Wolff B, Nyanzi B, Katongole G, Ssesanga D, Ruberantwari A, Whitworth J (2005). Evaluation of a home-based voluntary counselling and testing intervention in rural Uganda. Health Policy Plan.

[26] Day S, Lakhani D, Hankins M, Rodgers CA (2004). Improving uptake of HIV testing in patients with a confirmed STI. Int J STD AIDS.

[27] Mahto M, Higgins SP (2004). Increased uptake of HIV screening following introduction of “opt out” testing and results by telephone. Sex Transm Infect.

[28] Mermin J, Bunnell R, Lule J (2005). Developing an evidence-based, preventive care package for persons with HIV in Africa. Trop Med Int Health.

[29] Rotheram-Borus MJ, Leibowitz AA, Etzel MA (2006). Routine, rapid HIV testing. AIDS Educ Prev.

[30] Corbett EL, Dauya E, Matambo R (2006). Uptake of workplace HIV counselling and testing: a cluster-randomised trial in Zimbabwe. PLoS Med.

[31] Burke Johnson R, Onwuegbuzie AJ (2004). Mixed Methods Research: A Research Paradigm Whose Time Has Come. Educational Researcher.

[32] Lugada E, Levin J, Abang B (2010). Comparison of home and clinic-based HIV testing among household members of persons taking antiretroviral therapy in Uganda: results from a randomized trial. J Acquir Immune Defic Syndr.

[33] Sweat M, Morin S, Celentano D (2011). Community-based intervention to increase HIV testing and case detection in people aged 16-32 years in Tanzania, Zimbabwe, and Thailand (NIMH Project Accept, HPTN 043): a randomised study. Lancet Infect Dis.

[34] Callaghan M, Ford N, Schneider H (2010). A systematic review of task- shifting for HIV treatment and care in Africa. Hum Resour Health.

[35] Pavie J, Rachline A, Loze B (2010). Sensitivity of five rapid HIV tests on oral fluid or finger-stick whole blood: a real-time comparison in a healthcare setting. PLoS One.

[36] Chen MY, Bilardi JE, Lee D, Cummings R, Bush M, Fairley CK (2010). Australian men who have sex with men prefer rapid oral HIV testing over conventional blood testing for HIV. Int J STD AIDS.

[37] Choko A, Desmond N, Webb E Feasibility, Accuracy, and
Acceptability of Using Oral HIV Test Kits for Supervised
Community-level Self-testing in a Resource-poor High-HIV
Prevalence Setting: Blantyre, Malawi.

[38] Coetzee D, Hilderbrand K, Goemaere E, Matthys F, Boelaert M (2004). Integrating tuberculosis and HIV care in the primary care setting in South Africa. Trop Med Int Health.

[39] van der Merwe K, Chersich MF, Technau K, Umurungi Y, Conradie F, Coovadia A (2006). Integration of antiretroviral treatment within antenatal care in Gauteng Province, South Africa. J Acquir Immune Defic Syndr.

[40] April MD (2010). Rethinking HIV exceptionalism: the ethics of opt-out HIV testing in sub-Saharan Africa. Bull World Health Organ.

[41] Leon N, Naidoo P, Mathews C, Lewin S, Lombard C (2010). The impact of provider-initiated (opt-out) HIV testing and counseling of patients with sexually transmitted infection in Cape Town, South Africa: a controlled trial. Implement Sci.

[42] Nakibinge S, Maher D, Katende J, Kamali A, Grosskurth H, Seeley J (2009). Community engagement in health research: two decades of experience from a research project on HIV in rural Uganda. Trop Med Int Health.

[43] Beaudoin CE (2007). HIV prevention in sub-Saharan Africa: a multilevel analysis of message frames and their social determinants. Health Promot Int.

[44] Johnson D, Flora JA, Rimal RN (1997). HIV/AIDS public service announcements around the world: a descriptive analysis. J Health Commun.

[45] Lugada E, Millar D, Haskew J (2010). Rapid implementation of an integrated large-scale HIV counseling and testing, malaria, and diarrhea prevention campaign in rural Kenya. PLoS One.

[46] Anderson DA, Crowe SM, Garcia M (2011). Point-of-care testing. Curr HIV/AIDS Rep.

[47] Gray RH, Makumbi F, Serwadda D (2007). Limitations of rapid HIV-1 tests during screening for trials in Uganda: diagnostic test accuracy study. BMJ.

[48] Anzala O, Sanders EJ, Kamali A (2008). Sensitivity and specificity of HIV rapid tests used for research and voluntary counselling and testing. East Afr Med J.

[49] Kagulire SC, Opendi P, Stamper PD (2011). Field evaluation of five rapid diagnostic tests for screening of HIV-1 infections in rural Rakai, Uganda. Int J STD AIDS.

[50] Ma ZM, Stone M, Piatak  M (2009). High specific infectivity of plasma virus from the pre-ramp-up and ramp-up stages of acute simian immunodeficiency virus infection. J Virol.

[51] Pao D, Fisher M, Hue S (2005). Transmission of HIV-1 during primary infection: relationship to sexual risk and sexually transmitted infections. AIDS.

[52] Sagar M (2010). HIV-1 transmission biology: selection and characteristics of infecting viruses. J Infect Dis.

[53] Wawer MJ, Gray RH, Sewankambo NK (2005). Rates of HIV-1 transmission per coital act, by stage of HIV-1 infection, in Rakai, Uganda. J Infect Dis.

[54] Pilcher CD, Tien HC, Eron  JJ (2004). Brief but efficient: acute HIV infection and the sexual transmission of HIV. J Infect Dis.

[55] Fidler S, Fox J, Porter K, Weber J (2008). Primary HIV infection: to treat or not to treat?. Curr Opin Infect Dis.

[56] Sagar M, Laeyendecker O, Lee S (2009). Selection of HIV variants with signature genotypic characteristics during heterosexual transmission. J Infect Dis.

[57] Hayes RJ, White RG (2006). Amplified HIV transmission during early-stage infection. J Infect Dis.

[58] Everett DB, Baisely KJ, McNerney R (2010). Association of schistosomiasis with false-positive HIV test results in an African adolescent population. J Clin Microbiol.

[59] Beelaert G, Fransen K (2010). Evaluation of a rapid and simple fourth-generation HIV screening assay for qualitative detection of HIV p24 antigen and/or antibodies to HIV-1 and HIV-2. J Virol Methods.

[60]  Juusola L, Brandeau ML, Long EF, Owens DK, Bendavid E (2011). The Cost-Effectiveness of Symptom-Based Testing and Routine Screening for Acute HIV Infection in Men Who Have Sex with Men in the United States. AIDS.

[61] Obermeyer CM, Osborn M (2007). The utilization of testing and counseling for HIV: a review of the social and behavioral evidence. Am J Public Health.

[62] Koo DJ, Begier EM, Henn MH, Sepkowitz KA, Kellerman SE (2006). HIV counseling and testing: less targeting, more testing. Am J Public Health.

[63] Branson BM, Handsfield HH, Lampe MA (2006). Revised recommendations for HIV testing of adults, adolescents, and pregnant women in health-care settings. MMWR Recomm Rep.

[64] Matambo R, Dauya E, Mutswanga J (2006). Voluntary counseling and testing by nurse counselors: what is the role of routine repeated testing after a negative result?. Clin Infect Dis.

[65] Denison JA, O'Reilly KR, Schmid GP, Kennedy CE, Sweat MD (2008). HIV voluntary counseling and testing and behavioral risk reduction in developing countries: a meta-analysis, 1990-2005. AIDS Behav.

[66] Corbett EL, Makamure B, Cheung YB (2007). HIV incidence during a cluster-randomized trial of two strategies providing voluntary counselling and testing at the workplace, Zimbabwe. AIDS.

[67] World Health Organization (2005). Scaling-up HIV testing and counseling services: A toolkit for programme managers. WHO. Available at: www.who.int/hiv/pub/vct/counsellingtestingtoolkit.pdf.

[68] World Health Organization (2010). Antiretroviral therapy for HIV
infection in adults and adolescents. Recommendations for a public
health approach. 2010 revision. WHO. Available at: http://www.who.int/hiv/pub/arv/adult2010/en/index.html.

[69] Walensky RP, Paltiel AD, Losina E (2010). Test and treat DC: forecasting the impact of a comprehensive HIV strategy in Washington DC. Clin Infect Dis.

[70] waqqas s (2010). PlusNews. SOUTH AFRICA: National HIV testing campaign disappoints. PlusNews.

[71] Posse M, Meheus F, van Asten H, van der Ven A, Baltussen R (2008). Barriers to access to antiretroviral treatment in developing countries: a review. Trop Med Int Health.

[72] Decroo T, Telfer B, Biot M (2011). Distribution of Antiretroviral Treatment Through Self-Forming Groups of Patients in Tete Province, Mozambique. J Acquir Immune Defic Syndr.

[73] Jaffar S, Amuron B, Foster S (2009). Rates of virological failure in patients treated in a home-based versus a facility-based HIV-care model in Jinja, southeast Uganda: a cluster-randomised equivalence trial. Lancet.

[74] Medley A, Garcia-Moreno C, McGill S, Maman S (2004). Rates barriers and outcomes of HIV serostatus disclosure among women in developing countries: implications for prevention of mother-to-child transmission programmes. Bull World Health Organ.

[75] Howard AA, El-Sadr WM (2010). Integration of tuberculosis and HIV services in sub-Saharan Africa: lessons learned. Clin Infect Dis.

[76] Tudor Car L, van-Velthoven MH, Brusamento S (2011). Integrating prevention of mother-to-child HIV transmission (PMTCT) programmes with other health services for preventing HIV infection and improving HIV outcomes in developing countries. Cochrane Database Syst Rev.

[77] O'Brien D, Greig J, Sabapathy K, Shanks L Comparison of
integrated and vertical antiretroviral treatment programme
outcomes in nine countries in Sub-Saharan Africa.

[78] Muller M, Wandel S, Colebunders R, Attia S, Furrer H, Egger M (2010). Immune reconstitution inflammatory syndrome in patients starting antiretroviral therapy for HIV infection: a systematic review and meta-analysis. Lancet Infect Dis.

[79] Kitahata MM, Gange SJ, Abraham AG (2009). Effect of early versus deferred antiretroviral therapy for HIV on survival. N Engl J Med.

[80] Sterne JA, May M, Costagliola D (2009). Timing of initiation of antiretroviral therapy in AIDS-free HIV-1-infected patients: a collaborative analysis of 18 HIV cohort studies. Lancet.

[81] Shepherd BE, Jenkins CA, Rebeiro PF (2010). Estimating the optimal CD4 count for HIV-infected persons to start antiretroviral therapy. Epidemiology.

[82] Jain V, Deeks SG (2010). When to start antiretroviral therapy. Curr HIV/AIDS Rep.

[83] Siegfried N, Uthman OA, Rutherford GW (2010). Optimal time for initiation of antiretroviral therapy in asymptomatic, HIV-infected, treatment-naive adults. Cochrane Database Syst Rev.

[84] Zolopa A, Andersen J, Powderly W (2009). Early antiretroviral therapy reduces AIDS progression/death in individuals with acute opportunistic infections: a multicenter randomized strategy trial. PLoS One.

[85] Williams BG, Hargrove JW, Humphrey JH (2010). The benefits of early treatment for HIV. AIDS.

[86] US National Institutes of Health (2011). Strategic Timing of Antiretroviral
Treatment. Clinical Trials Feeds, updated. Available
at: http://clinicaltrialsfeeds.org/clinical-trials/show/NCT00867048.

[87] Nachega JB, Trotta MP, Nelson M, Ammassari A (2009). Impact of metabolic complications on antiretroviral treatment adherence: clinical and public health implications. Curr HIV/AIDS Rep.

[88] Izzedine H, Harris M, Perazella MA (2009). The nephrotoxic effects of HAART. Nat Rev Nephrol.

[89] D'Andrea G, Brisdelli F, Bozzi A (2008). AZT: an old drug with new perspectives. Curr Clin Pharmacol.

[90] van Leth F, Andrews S, Grinsztejn B (2005). The effect of baseline CD4 cell count and HIV-1 viral load on the efficacy and safety of nevirapine or efavirenz-based first-line HAART. AIDS.

[91] Watts DH (2007). Teratogenicity risk of antiretroviral therapy in pregnancy. Curr HIV/AIDS Rep.

[92] Hsu H, Rydzak C, Cotich K (2011). Quantifying the risks and benefits of efavirenz use in HIV-infected women of childbearing age in the USA. HIV Med.

[93] Ford N, Mofenson L, Kranzer K (2010). Safety of efavirenz in first-trimester of pregnancy: a systematic review and meta-analysis of outcomes from observational cohorts. AIDS.

[94] Campaign for Access to Essential Medicines (2011). Untangling the Web
of Antiretroviral Price Reductions. Available at: http://utw.msfaccess.org/.

[95] Peter T, Blair D, Reid M, Justman J (2010). DART and laboratory monitoring of HIV treatment. Lancet.

[96] BiotechBistro Forums Zyomyx point-of-care CD4 test selected by CD4 initiative to help treat HIV/AIDS. Avaiable at: http://biotechbistro.com/forum/topic/zyomyx-point-of-care-cd4-
test-selected-by-cd4-initiative-to-help-treat-hivaids.

[97] Pham PA (2009). Antiretroviral adherence and pharmacokinetics: review of their roles in sustained virologic suppression. AIDS Patient Care STDs.

[98] Bartlett JA, Buda JJ, von Scheele B (2006). Minimizing resistance consequences after virologic failure on initial combination therapy: a systematic overview. J Acquir Immune Defic Syndr.

[99] El-Sadr WM, Lundgren JD, Neaton JD (2006). CD4+ count-guided interruption of antiretroviral treatment. N Engl J Med.

[100] Lundgren JD, Babiker A, El-Sadr W (2008). Inferior clinical outcome of the CD4+ cell count-guided antiretroviral treatment interruption strategy in the SMART study: role of CD4+ Cell counts and HIV RNA levels during follow-up. J Infect Dis.

[101] Emery S, Neuhaus JA, Phillips AN (2008). Major clinical outcomes in antiretroviral therapy (ART)-naive participants and in those not receiving ART at baseline in the SMART study. J Infect Dis.

[102] Burman W, Grund B, Neuhaus J (2008). Episodic antiretroviral therapy increases HIV transmission risk compared with continuous therapy: results of a randomized controlled trial. J Acquir Immune Defic Syndr.

[103] Rieder P, Joos B, von Wyl V (2010). HIV-1 transmission after cessation of early antiretroviral therapy among men having sex with men. AIDS.

[104] Das M, Chu PL, Santos GM (2010). Decreases in community viral load are accompanied by reductions in new HIV infections in San Francisco. PLoS One.

[105] Potter D, Goldenberg RL, Chao A (2008). Do targeted HIV programs improve overall care for pregnant women?: Antenatal syphilis management in Zambia before and after implementation of prevention of mother-to-child HIV transmission programs. J Acquir Immune Defic Syndr.

[106] Fideli US, Allen SA, Musonda R, Trask S, Hahn BH, Weiss H (2001). Virologic and immunologic determinants of heterosexual transmission of human immunodeficiency virus type 1 in Africa. AIDS Res Hum Retroviruses.

[107] Tovanabutra S, Robison V, Wongtrakul J (2002). Male viral load and heterosexual transmission of HIV-1 subtype E in northern Thailand. J Acquir Immune Defic Syndr.

[108] Vergidis PI, Falagas ME, Hamer DH (2009). Meta-analytical studies on the epidemiology, prevention, and treatment of human immunodeficiency virus infection. Infect Dis Clin North Am.

[109] Donnell D, Baeten JM, Kiarie J (2010). Heterosexual HIV-1 transmission after initiation of antiretroviral therapy: a prospective cohort analysis. Lancet.

[110] Graham SM, Holte SE, Peshu NM (2007). Initiation of antiretroviral therapy leads to a rapid decline in cervical and vaginal HIV-1 shedding. AIDS.

[111] Fiore JR, Suligoi B, Saracino A (2003). Correlates of HIV-1 shedding in cervicovaginal secretions and effects of antiretroviral therapies. AIDS.

[112] Nagot N, Ouedraogo A, Weiss HA (2008). Longitudinal effect following initiation of highly active antiretroviral therapy on plasma and cervico-vaginal HIV-1 RNA among women in Burkina Faso. Sex Transm Infect.

[113] Burns DN, Dieffenbach CW, Vermund SH (2010). Rethinking prevention of HIV type 1 infection. Clin Infect Dis.

[114] Walensky RP, Ciaranello AL, Park JE, Freedberg KA (2010). Cost-effectiveness of laboratory monitoring in sub-Saharan Africa: a review of the current literature. Clin Infect Dis.

[115] Hayes RJ, Moulton LH (2009). Cluster randomised trials.

[116] Hayes RJ, Alexander NDE, Bennett S, Cousens SN (2000). Design and analysis issues in cluster-randomized trials of interventions against infectious diseases. Stat Methods Medical Res.

[117] Kurth AE, Celum C, Baeten JM, Vermund SH, Wasserheit JN (2011). Combination HIV prevention: significance, challenges, and opportunities. Curr HIV/AIDS Rep.

